# P66Shc expression in diabetic rat retina

**DOI:** 10.1186/s12886-018-0724-3

**Published:** 2018-02-27

**Authors:** Ming-Hui Zhao, Jianyan Hu, Shufeng Li, Qiang Wu, Peirong Lu

**Affiliations:** 1 Department of Ophthalmology, The First Affiliated Hospital of Soochow University, Suzhou, 215006 China; 2 Department of Ophthalmology, Shanghai Jiaotong University Affiliated Sixth People’s Hospital, Shanghai, 200233 China

**Keywords:** Diabetic retinopathy, p66Shc, reactive oxygen species, Oxidative stress, Apoptosis

## Abstract

**Background:**

P66Shc is partially localised within the mitochondrial fraction. It is primarily related to the generation of mitochondrial reactive oxygen species and apoptosis. Based on previous studies, we hypothesize that in the retina, p66Shc may exist and affect the development of diabetic retinopathy. The purpose of this study was to investigate p66Shc expression in retinal in streptozotocin-induced diabetic (SD) rats, which may provide a pathway to study the pathogenesis of diabetic retinopathy.

**Methods:**

Reverse transcription-polymerase chain reaction (RT-PCR) and western blot were used to detect retinal p66Shc mRNA and protein expression in SD rats, respectively. Immunohistochemical staining was applied to detect the location of rat retinal p66Shc expression. TUNEL assay was applied to detect the number of apoptotic cells.

**Results:**

P66Shc expression was found in the retina of normal and diabetic rats, and the level of mRNA and protein expression increased with the progression of diabetes mellitus (DM). P66Shc expression was mainly located in the retinal ganglion cell layer and inner nuclear layer. Compared with the normal group, retinal cell tissue apoptosis rate in the D12w group was significantly increased.

**Conclusion:**

Rat retinal p66Shc expression was mainly in the ganglion cell layer and inner nuclear layer. As the degree of DM progressed, p66Shc expression gradually increased, and the number of apoptotic cells also increased.

## Background

Diabetic retinopathy (DR) is one of the most common complications in both type 1 and type 2 diabetes mellitus (DM). Through a series of mechanisms, such as protein kinase-C activation [1, 2], polyol pathway hyperfunction [3], and the hexosamine pathway [3], reactive oxygen species (ROS) and the advanced glycation end products are generated by hyperglycaemic conditions, which will affect the retinal microvascular structure [4, 5]. Among the spectrum of biochemical changes induced by high-glucose conditions, ROS generation seems to be one of the main pathophysiological mechanisms [6]. Because the eyes constantly exposure to sunlight, atmospheric oxygen, and environmental chemicals, ocular tissues are prone to ROS damage. In addition, free radical-catalysed peroxidation of long-chain polyunsaturated fatty acids (LCPUFAs) will generate LCPUFA metabolites, including isoprostanes and neuroprostanes, which may further exert toxicological and pharmacological actions in ocular tissues [7]. The precise location and the exact mechanism of physiologically relevant ROS generation within the respiratory chain have not yet been determined, however, it is generally believed that the major site of superoxide production is the respiratory chain in mitochondria. The increase of ROS will activate oxidative stress, and lead to apoptosis. Studies of rats exposed to streptozotocin (STZ) revealed that the apoptosis-specific changes in retinal ganglion cells were observed as early as after 1 month of diabetesspecific metabolic disorders [8, 9]. Noticeably, these changes were not associated with diabetic retinopathy-specific vascular injury [8, 10], which means that the apoptosis is an early pathological feature of DR. Biochemical pathways of apoptosis activation can be extra- or intracellular, and caspase-dependent or caspase-independent [11]. So reducing the extracellular apoptosis inducing signal or interfere with the intracellular apoptosis related signal pathway is important for the prevention and treatment of DR [12, 13].

Adaptor protein p66Shc is a newly recognised mediator of mitochondrial dysfunction. It is expressed in most mammalian tissues. Three isoforms are derived by alternative splicing from the *Shc* locus: p46Shc, p52Shc, and p66Shc [14]. These proteins have 3 same functional domains in structure: a collagen-homology region, Src-homology 2 domain, and a phosphotyrosine-binding domain [14]. Compared with the other two kinds of protein, P66Shc has an additional N-terminal region, which is required for its redox activity [14]. This protein is mainly localised within the mitochondrial fraction, and is primarily associated with mitochondrial ROS generation and apoptosis [15]. Via the oxidation of cytochrome C, p66Shc utilises the mitochondrial electron transfer chain during the generation of hydrogen peroxide (H_2_O_2_) [16]. P66Shc is entered into the mitochondrial intermembrane space by the form of Ser36 phosphorylation (p-p66Shc) [17]. Former studies showed that p66Shc^−/−^ cells can decrease alterations of mitochondrial DNA, reduce intracellular ROS levels, and resistant apoptosis which was induced by a variety of stimuli, such as ultraviolet radiation, H_2_O_2_, hypoxia/reoxygenation, and human immunodeficiency virus-1 [17, 18]. Similarly, in p66Shc^−/−^ mice also found increased resistance to oxidative stress and a prolonged life span [18]. They are also prevented the development of diabetic glomerulopathy, possibly by blocking the production of hyperglycaemia-induced ROS [19]. In Graiani et al.’s study, they found that in the ischaemia-reperfusion injury model, mitochondrial dysfunction in renal proximal tubule cells was mediated by p66Shc, which induced apoptosis in myocardial cells [20].

Diabetic nephropathy and DR are the two most common forms of diabetic microvascular complications, and both diseases share a common pathogenesis. Based on these considerations, we postulated that p66Shc could also play a role in the development of DR. The aim of this study was to determine the expression of p66Shc in diabetic retinas.

## Methods

### Experimental animals

In this study, we used the 3-week-old fasted male Sprague Dawley (SD) rats, which were obtained from Shanghai Sippr-BK laboratory animal Co. Ltd. (Shanghai, China). The mean weight of the SD rats was approximately 150 g. Treatment of animals was compled with the rules of “Instruction and Administration of Experimental Animals”, and was approved by the Shanghai Jiaotong University affiliated No.6 hospital. Animals received an intraperitoneal streptozotocin injection (60 mg/kg dissolved in the citric acid solution, pH 4.5) on three successive days. 48 h after STZ injection, the rats with glucose levels > 250 mg/dl were defined as having DM. Non-diabetic age-matched control animals were injected with 0.01 M sodium citrate buffer. According to the DM course, all of the rats were assigned to 3 groups: 15 rats of D4w (4 weeks after diabetes onset) and 15 rats of D12w (12 weeks after diabetes onset) and 15 rats of not-diabetic control.

### Real-time PCR

We used the Trizol method to extract RNA. the primer of *p66Shc* was designed by The PRIMER PREMIER 5.0 software, and was synthesised by Invitrogen Biotechnology Co., LTD (Shanghai, China). Table 1 gives the PCR primer sequences. 1 ml Trizol (Invitrogen Life Technologies, Shanghai, China) was mixed with Retinal tissue, and completely homogenised. Then we put the mixture on ice for about 5 min, in order to allow full denaturation. Then it mixed with 0.25 ml chloroform, oscillated for 15 s and stood for 3 min. At 4 °C, the mixture was centrifuged at 13000 *g* for 8 min; then supernatant was obtained. After 0.5 ml isopropanol was added and mixed, the mixture stood again for about 10 min at room temperature. At 4 °C, the mixture was centrifuged at 13000 *g* for 10 min again, and the supernatant was removed. After washed and precipitated the pellet, we added 1.5 ml 75% ethanol, and centrifuged at 13000 *g* for 5 min at 4 °C. Then we removed supernatant, absorbed the most of the ethanol, precipitated the RNA, and air dried for 5–10 min. Using the RevertAid First Strand cDNA Synthesis Kit (Thermo, Shanghai, China) according to the manufacturer’s protocols, total RNA (2 μg) was reverse transcribed. The internal reference gene was *GAPDH*. 95 °C for 10 min, followed by 40 cycles of 95 °C for 15 s and 60 °C for 60 s, the 20 μl reaction mixtures were amplified. The data were analysed using the 2^-ΔΔCt^ method.

**Table 1:** The PCR primers

Primer	F/R	Sequcence (5′-3′)
GAPDH	F	TTCCTACCCCCAATGTATCCG
	R	CATGAGGTCCACCACCCTGTT
P66Shc	F	TGTCAATAAGCCCACACGAGG
	R	CTTCACACACCAAACTGATAGCCT

### Western blot

Retinas were dissected, harvested in radio-immunoprecipitation assay (RIPA) lysis buffer (Beyotime Biotechnology, China), homogenised, and centrifuged at 12000 g for 20 min at 4 °C. The protein sample extracted from the retina was buffered in the liquid, mixed well, and heated in hot water for 10 min, cooled in ice water immediately. We used sodium dodecyl sulphate (SDS)-polyacrylamide gel electrophoresis to conduct gel electrophoresis. Proteins (10–20 μg) were loaded onto an SDS 12% polyacrylamide gel. The proteins were transferred onto polyvinylidene fluoride membranes after electrophoresis. 5% skimmed milk in Tween/Tris-buffered saline (TBST) was used to block nonspecific binding. Membranes were incubated with primary antibodies against Shc (1:1000; Santa Cruz, Shanghai, China), and with β-actin (1:2000; KangChen Bio-tech, Shanghai, China) prepared in 5% skimmed milk in TBST overnight at 4 °C. The membrane was washed three times using phosphate buffer solution Tween (PBST) buffer for 15 min in a shaker. The membrane was immersed into the secondary antibody with horseradish peroxidase for 1 h at room temperature. Following three further 15-min washes of the membrane in a shaker using the PBST buffer, by using an enhanced chemiluminescence reaction kit according to the manufacturer’s instructions (Goodbio Biological Technology Co., LTD, Wuhan, China), protein bands were visualised and photographed with a Tanon 5500 Imager (Tanon, Shanghai, China). The bands were analysed with AlphaEaseFC (Alpha Innotech, USA). Each band was normalised against the corresponding β-actin band. Changes in expression of protein were expressed as the ratio of diabetic rats versus non-diabetic rats’ levels.

### Immunohistochemistry (IHC)

Using an abdominal injection of a 2% pentobarbital solution (30 mg/kg) (Goodbio Biological Technology Co., LTD, Wuhan, China), the rats were euthanized. Eyeballs were removed. Then the eyes soaked in 4% paraformaldehyde (phosphate buffer saline (PBS) buffered) for 24 h. In order to eliminate enzymatic activity, retinas were incubated in 3% H_2_O_2_ for 10 min and washed twice for 5 min using PBS. The samples were blocked using 5% goat serum (diluted in PBS) and incubated at room temperature for 10 min. The blocking serum was removed,primary antibody was added (anti-Shc antibody, 1:125 dilution, Santa Cruz, Shanghai, China), and then be incubated at 4 °C overnight. Retinas were washed twice with PBST, followed by reacting with the secondary immunoglobulin (Dako, Beijing, China) at 37 °C for 30 min. Immunoreactivity was visualised using a Horseradish Peroxidase Colour Development Kit (diaminobenzidine, Goodbio Biological Technology Co., LTD, Wuhan, China), followed by fully washing using running water, and covered with a cover slip. The samples without primary antibody added were as negative controls.

### TUNEL assay

In brief, Terminal Deoxynucleotidyl Transferase dUTP Nick End Labeling (TUNEL) assay kit (Roche Applied Science, Sweden) was used to detect tissues apoptosis. Paraffin sections from histological assessment were routinely de-paraffinized, rehydrated, and then rinsed by PBS. TUNEL reaction solution and Converter-POD were added after blocking endogenous peroxidise activity by H_2_O_2_ in methanol, permeability liquid (1 g/L Triton X-100 was dissolved in 0.1% sodium citrate). 3, 3- diaminobenzidine (DAB) stained each slice, and then cell apoptosis was observed under a microscope. Cells with brown granules in the nucleus were considered as apoptosis positive cells. Under high magnification field (200×), we randomly selected five fields in each slice. Apoptosis rate means the percentage of apoptosis positive cells relative to the total cell.

### Statistical analysis

Statistical analysis was performed using the Statistical Package for Social Sciences (version 11.0, SPSS Inc., Chicago, IL, USA). Data were expressed as the mean standard deviation (SD). The Student’s t-test was applied to analyse the difference between the diabetic and control groups. A value of *P* < 0.05 was considered to be statistically significant.

## Results

### RT-PCR

**Fig. 1:**
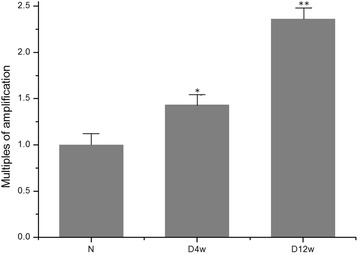
RT-PCR showed that the level of rat retinal p66Shc mRNA expression significantly increased over 12 weeks of diabetes mellitus (N: control group, D4w: 4 weeks after diabetes onset, D12w: 12 weeks after diabetes onset, *P < 0.05, **P < 0.01, compared to control)

Rat retinal p66Shc expression was detected by RT-PCR, and was found in the normal, D4w and D12w groups. With the progress of DM, p66Shc expression increased (*P* < 0.05 in D4w, and *P* < 0.01 in D12w, compared to normal group) (Fig. 1).

### Western blot

**Fig. 2:**
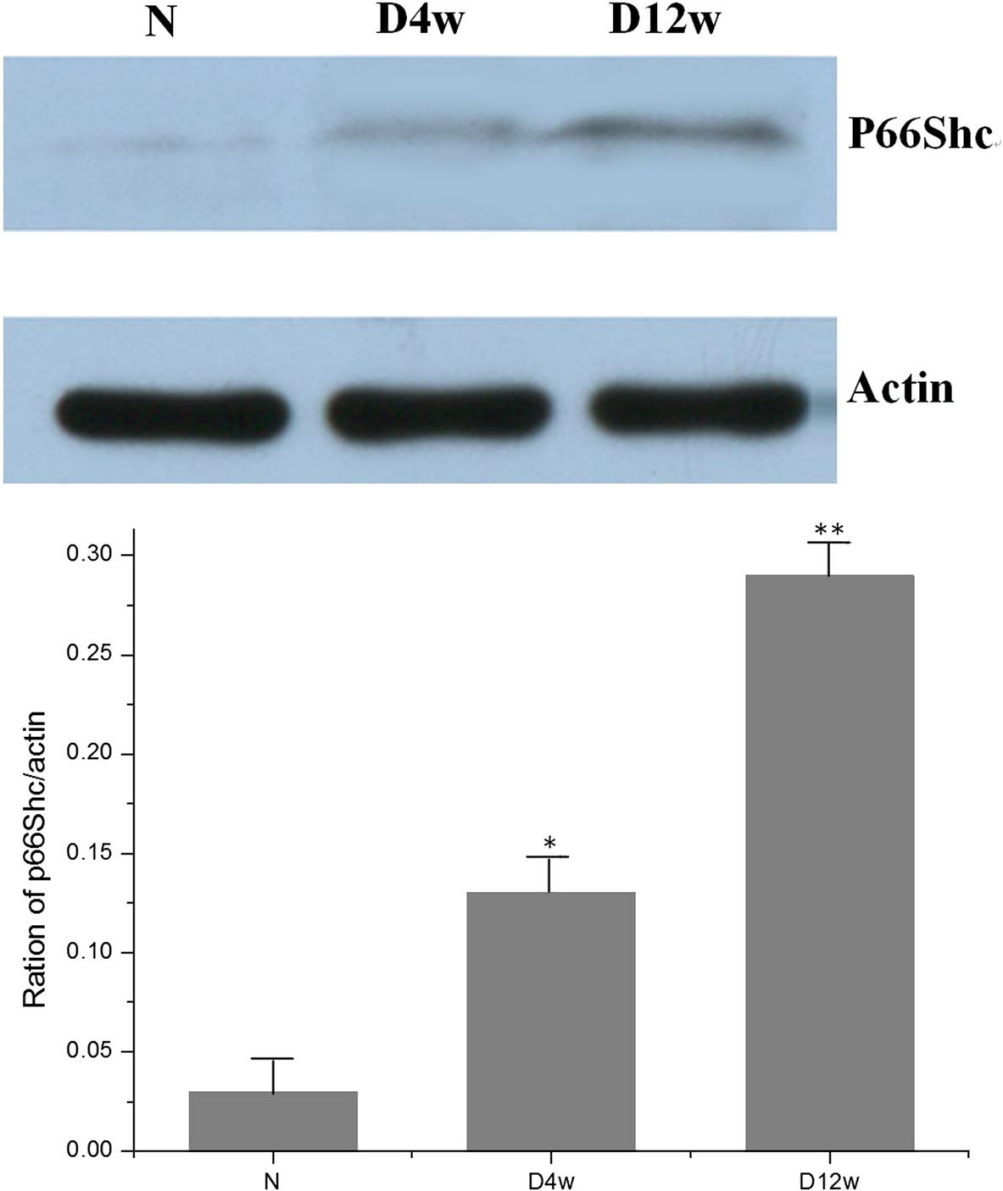
P66Shc protein expression significantly increased with the progression of diabetes mellitus (N: control group, D4w: 4 weeks after diabetes onset, D12w: 12 weeks after diabetes onset, *P < 0.05, ** *P* < 0.01, compared to control)

As for mRNA, protein expression was found in the normal, D4w and D12w groups. The expression level increased with the progress of DM (P < 0.05 in D4w, and *P* < 0.01 in D12w, compared to normal group) (Fig. 2).

### Immunohistochemistry (IHC)

**Fig. 3:**
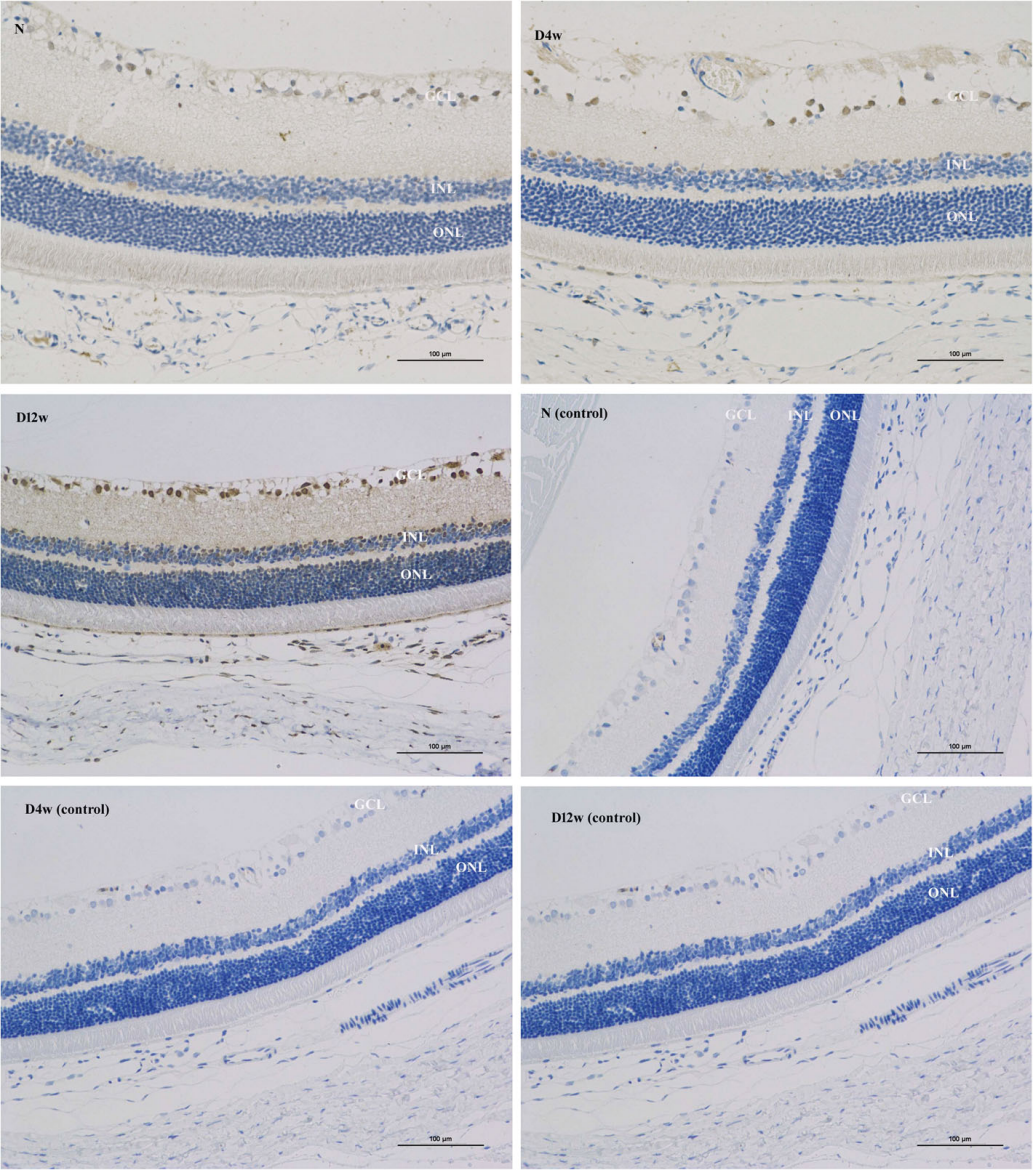
Immunohistochemical staining of frozen sections. P66Shc was expressed in the retina of normal rats and of rats with diabetes mellitus (DM). Greater p66Shc expression was found in the DM group compared to the control group. The expression increased with the progress of DM. (N: control group, D4w: 4 weeks after diabetes onset, D12w: 12 weeks after diabetes onset, control: the samples without primary antibody added)

P66Shc was mainly expressed in the ganglion cell layer (GCL) and inner nuclear layer (INL) of the retina in normal rats. With the progression of DM, the expression was increased. In the D12w group, p66Shc was expressed in all retinal layers, and was more concentrated in the GCL and INL (Fig. 3).

### Apoptosis analysis

**Fig. 4:**
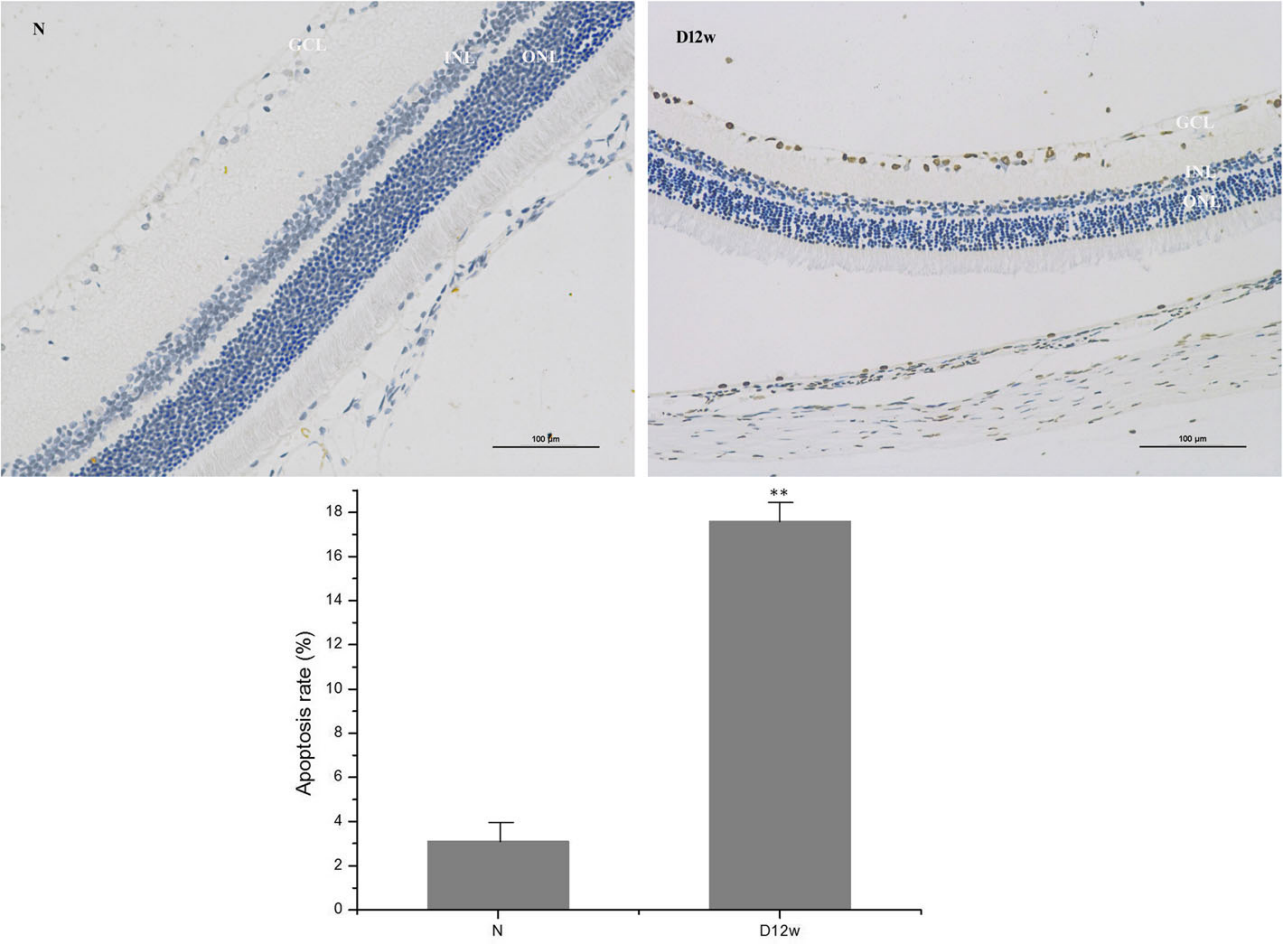
The detection of apoptotic cells by TUNEL assay for DNA Strand break labeling. TUNEL positive (brown nuclei) and negative cells were further counted for apoptosis rate. (N: control group; D12w: 12 weeks after diabetes onset, **P < 0.01, compared with control group)

Compared with the normal group, retinal cell tissue apoptosis rate in the D12w group was significantly increased (*P* < 0.01) (Fig. 4).

## Discussion

DM is one of the most common oxidative-stress related diseases. It seems that oxidative stress from the mitochondrial electron-transport chain and the excessive production of superoxide anion mediate hyperglycaemic damage [21]. Jain and colleagues found that in diabetic patients, peroxidation of membrane lipids was increased, and malonyldialdehyde in erythrocytes was accumulated. The possible reasons for these may be that during periods of poor metabolic control,the blood levels of ketone bodies increased [22], as well as the effect of increased levels of circulating cytokines, including tumour necrosis factor-α and interleukin-6 [23].

Our study followed the course of DM for 12 weeks in the SD rats to investigate whether p66Shc is expressed in the retina and what changes occur in DM. The results showed that p66Shc was expressed in the retina of normal and DM groups both at the mRNA and protein levels. Compared to the normal group, p66Shc expression in the DM groups was statistically increased, which suggested that there was some degree of relationship between the course of the disease and the expression of p66Shc. This was similar to previous studies of p66Shc expression in other diseases. Earlier studies suggested that p66Shc is a vital adaptor protein that regulates oxidative stress and life span in mammalian cells. Genetic deletion of p66Shc attenuated hyperglycaemia-induced endothelial dysfunction and oxidative damage [24, 25]. In ventricular pacing-induced cardiomyopathy dog models, a progressive p66Shc overexpression was induced by increased ROS production and mitochondrial dysfunction, which was correlated with cytochrome c release, parameters of ventricular dysfunction, and activation of procaspases [26]. P66Shc expression and activity are significantly increased in the kidneys of SD rats and db/db mice (a type 2 diabetic mouse model) [27]. In contrast, P66-null Akita mice display marked attenuation of oxidative stress and glomerular/tubular injury and a distinct reduction in urine albumin excretion [27]. The deletion of p66Shc also reduced tissue damage [28] and vascular cell apoptosis [29], as well as protect against ROS-mediated, age-dependent endothelial dysfunction [30]. All these studies suggested that p66Shc is associated with increased oxidative damage [31]. Intracellular ROS availability was increased by P66Shc induction, which in turn affects the rate of oxidative damage.

The p66Shc expression site in the rat retina was detected by frozen-section immunohistochemical staining. It was observed that in normal rat retinas, p66Shc was expressed, mainly in the GCL and INL. As the progression of disease, the expression increased, and in the D12w group, p66Shc was observed in all retinal layers, mainly in the GCL and INL. TUNEL assay suggested that with the progression of DM, the number of apoptotic cells increased. Barber et al. [32] analyzed paraffin-embedded retinal specimens from STZ-exposed rats, obtained after 30 weeks of experimentally induced diabetes, and found that a 10% reduction in the total number of retinal ganglion cells, along with a 22% decrease in the thickness of the inner ganglion layer of the retina, and a 14% decrease in the thickness of the inner nuclear layer. Interestingly, no changes in the thickness of the outer ganglion cell layer, which suggested that the processes of apoptosis are more intense within the inner layers of the retina [32]. Oxidative stress activates stress-related signalling to trigger apoptosis [33]. Diabetic glomerulopathy in wild-type mice without direct intervention (e.g. STZ injection) was associated with cell-death rate and enhanced extracellular matrix protein expression. Renal p66Shc mRNA and protein levels also increased in diabetic wild-type mice [34, 35]. On the contrary, no increase in the glomerular cell-death rate was found in diabetic p66Shc knockout (KO) mice, meanwhile, compared to wild-type diabetic mice, less marked matrix deposition was found in KO mice. ROS levels, glucose-induced apoptosis, and upregulation of extracellular matrix were no or significantly attenuated in mesangial cells from KO mice, which supporting the concept that p66Shc protein deficiency was associated with reduced susceptibility to diabetes-induced oxidative stress, attenuated changes in cell turnover and matrix, and reduced apoptosis [19].

P66Shc expression is regulated by multiple factors. For example, its phosphorylation is regulated by adaptor protein Eps8 together with E3b1; meanwhile Rac1 can reduce ubiquitylation and increase the stability of p66Shc protein [36]. Sos1 and Eps8/E3b1 form a complex, which can activate Rac1 [36]. P66Shc can detach Sos1 from the growth factor receptor-bound protein 2 (Grb2)/Sos1 pool and transfer it with the Eps8/E3b1 pool, which activate Rac1 and result increased generation of oxidants [37]. During severe oxidative stress, the combination of p66Shc with activated epidermal growth factor receptor (EGFR) and Grb2 increased. This binding results in the separation of the Sos1 adaptor protein from the EGFR-recruited signalling complex. Ras/MEK/ERK (extracellular-signal regulated kinase) activation was terminated [38]. Conversely, p66Shc is tyrosine phosphorylated by receptor tyrosine kinases which were stimulated by growth factors. It is unable to activate the Ras-MAPK-Fos pathways after the tyrosine-phosphorylated P66Shc binds Grb2 [18]. The Ras signalling pathway is also inhibited when p66Shc is over-expressed by the stimulation of cytokines or growth factors [37]. By insulin growth factor, p66Shc inhibit stimulation of the MEK (MAPK-ERK Kinase)/ERK pathway as well.

In general, p66Shc is implicated in receptor tyrosine kinase signal transduction, and is classically known as a signalling protein. By Ser36 phosphorylation of the protein, p66Shc plays a role in accumulation of intracellular ROS. It has been regarded as a longevity protein and a sensor of oxidative stress-induced apoptosis in mammals. P66Shc expression and/or function changes may play an important role in the pathogenesis of type 2 diabetes, so it may be an effective target for the treatment of DR.

This study has shown that there was p66Shc expression in rat retinal tissues, and the expression was increased along with the progression of DM. These results may offer some explanation about the relationship between p66Sch and DR, and help to understand the mechanism of DR, which may potentially allow its treatment at an earlier stage. Further studies are still required in the p66Shc-specific functional mechanism in DR.

## Conclusion

Rat retinal p66Shc expression was mainly in the ganglion cell layer and inner nuclear layer. As the degree of DM progressed, p66Shc expression gradually increased, and the number of apoptotic cells also increased.

## Abbreviations

DM: Diabetes mellitus
DR: Diabetic retinopathy
IHC: Immunohistochemistry
LCPUFAs: Long-chain polyunsaturated fatty acids
PBS: Phosphate buffer saline
PBST: Phosphate buffer solution Tween
RIPA: Radio-immunoprecipitation assay
ROS: Reactive oxygen species
RT-PCR: Reverse transcription-polymerase chain reaction
SD: Streptozotocin-induced diabetic
STZ: Streptozotocin
TBST: Tween/Tris-buffered saline
TUNEL: Terminal Deoxynucleotidyl Transferase dUTP Nick End Labeling
