# Correction to: P66Shc expression in diabetic rat retina

**DOI:** 10.1186/s12886-019-1071-8

**Published:** 2019-03-01

**Authors:** Ming-Hui Zhao, Jianyan Hu, Shufeng Li, Qiang Wu, Peirong Lu

**Affiliations:** 1 Department of Ophthalmology, The First Affiliated Hospital of Soochow, University, Suzhou, 215006 China; 2 Department of Ophthalmology, Shanghai Jiaotong University Affiliated Sixth People’s Hospital, Shanghai, 200233 China

**Correction to:** **BMC Ophthalmol.** **2018;18(1):58**

**https://doi.org/10.1186/s12886-018-0724-3**

Following publication of the original article [1], the authors notified us of an error in Fig. 3. In this correction article, D12w (control) was updated.

**Fig. 3:**
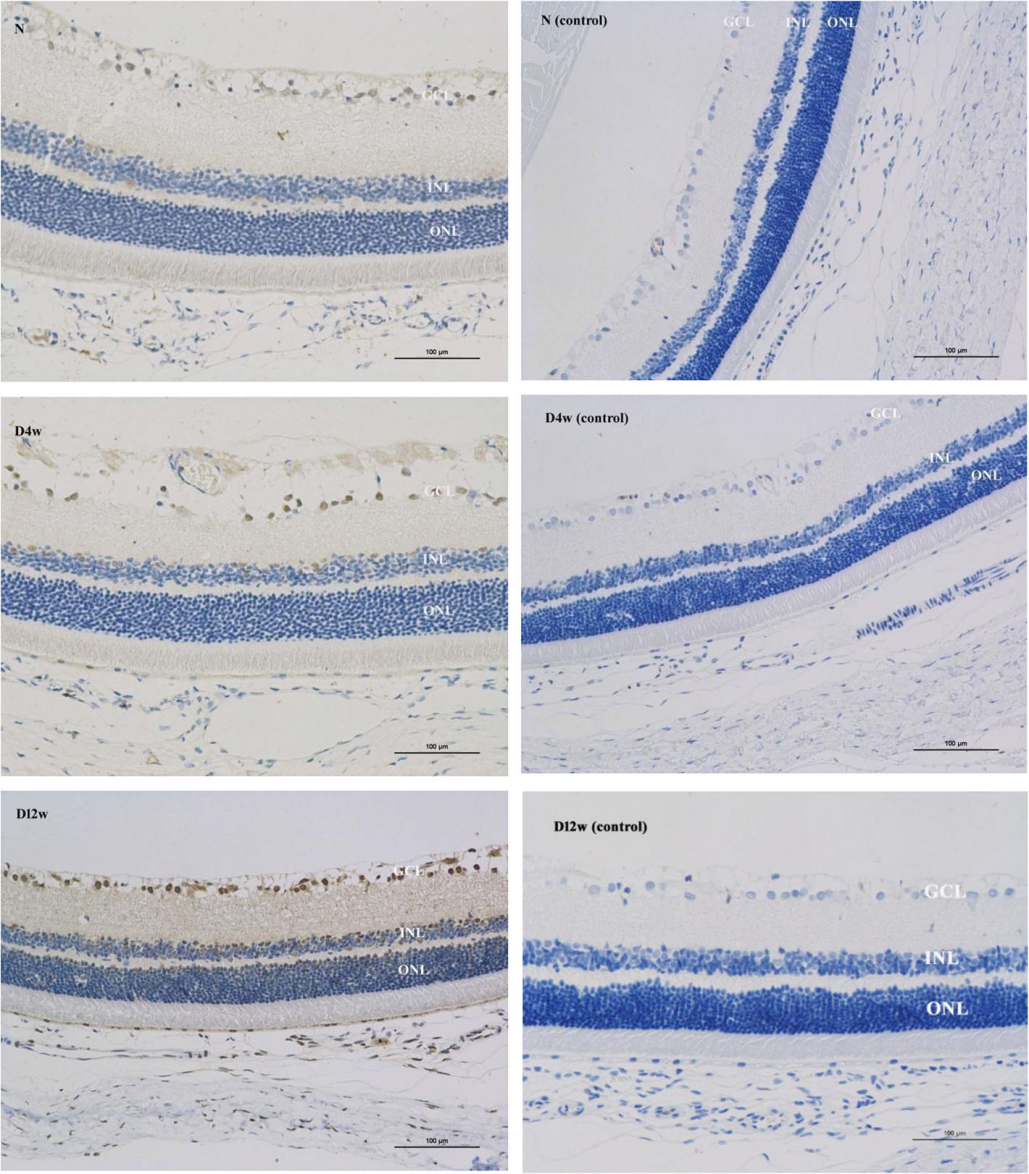
Immunohistochemical staining of frozen sections. P66Shc was expressed in the retina of normal rats and of rats with diabetes mellitus (DM). Greater p66Shc expression was found in the DM group compared to the control group. The expression increased with the progress of DM. (N: control group, D4w: 4 weeks after diabetes onset, D12w: 12 weeks after diabetes onset, control: the samples without primary antibody added)
